# Identification of traumatic acid as a potential plasma biomarker for sarcopenia using a metabolomics‐based approach

**DOI:** 10.1002/jcsm.12895

**Published:** 2021-12-22

**Authors:** Jaw‐Shiun Tsai, San‐Yuan Wang, Chin‐Hao Chang, Chin‐Ying Chen, Chiung‐Jung Wen, Guan‐Yuan Chen, Ching‐Hua Kuo, Y. Jane Tseng, Ching‐Yu Chen

**Affiliations:** ^1^ Department of Family Medicine National Taiwan University Hospital, National Taiwan University Taipei Taiwan; ^2^ Department of Family Medicine, College of Medicine National Taiwan University Taipei Taiwan; ^3^ Master Program in Clinical Genomics and Proteomics, College of Pharmacy Taipei Medical University Taipei Taiwan; ^4^ Department of Medical Research National Taiwan University Hospital Taipei Taiwan; ^5^ Department of Geriatrics and Gerontology National Taiwan University Hospital Taipei Taiwan; ^6^ Department and Graduate Institute of Forensic Medicine, College of Medicine National Taiwan University Taipei Taiwan; ^7^ The Metabolomics Core Laboratory, Center of Genomic Medicine National Taiwan University Taipei Taiwan; ^8^ School of Pharmacy, College of Medicine National Taiwan University Taipei Taiwan; ^9^ Department of Pharmacy National Taiwan University Hospital, National Taiwan University Taipei Taiwan; ^10^ Department of Computer Science and Information Engineering National Taiwan University Taipei Taiwan; ^11^ Graduate Institute of Biomedical Electronics and Bioinformatics National Taiwan University Taipei Taiwan

**Keywords:** Sarcopenia, Elderly, Metabolomics, Traumatic acid

## Abstract

**Background:**

The pathogenesis of sarcopenia is complex and has not been well explored. Identifying biomarkers is a promising strategy for exploring the mechanism of sarcopenia. This study aimed to identify potential biomarkers of sarcopenia through a metabolomic analysis of plasma metabolites in elderly subjects (≥65 years of age) vs. younger adults (<65 years of age).

**Methods:**

Of the 168 candidates in the Comprehensive Geriatric Assessment and Frailty Study of Elderly Outpatients, 24 elderly subjects (≥65 years of age) with sarcopenia were age and sex matched with 24 elderly subjects without sarcopenia. In addition, 24 younger adults were recruited for comparison. Muscle strength, gait speed, and metabolic and inflammatory parameters, including plasma tumour necrosis factor‐α, C‐reactive protein, irisin, and growth differentiation factor 15 (GDF‐15) levels were assessed. Metabolomic analysis was carried out using the plasma metabolites.

**Results:**

Seventy‐two participants were enrolled, including 10 (41.6%) men and 14 (58.3%) women in both groups of elderly subjects. The median ages of elderly subjects with and without sarcopenia were 82 (range: 67–88) and 81.5 (range: 67–87) years, respectively. Among the 242 plasma metabolic peaks analysed among these three groups, traumatic acid was considered as a sarcopenia‐related metabolite. The plasma traumatic acid signal intensity level was significantly higher in elderly subjects with sarcopenia than in elderly subjects without sarcopenia [591.5 (inter‐quartile range, IQR: 491.5–664.5) vs. 430.0 (IQR: 261.0–599.5), *P* = 0.0063]. The plasma concentrations of traumatic acid were 15.8 (IQR: 11.5–21.7), 21.1 (IQR: 16.0–25.8), and 24.3 (IQR: 18.0–29.5) ppb in younger adults [age range: 23–37 years, 12 (50%) men], elderly subjects without sarcopenia, and elderly subjects with sarcopenia, respectively, thereby depicting an increasing tendency (*P* for trend = 0.034). This pattern was similar to that of GDF‐15, a recognized sarcopenia‐related factor. Plasma traumatic acid concentrations were also positively correlated with the presence of hypertension (*r* = 0.25, *P* = 0.034), glucose AC (*r* = 0.34, *P* = 0.0035), creatinine (*r* = 0.40, *P* = 0.0006), and GDF‐15 levels (*r* = 0.25, *P* = 0.0376), but negatively correlated with the Modification of Diet in Renal Disease‐simplify‐glomerular filtration rate (*r* = −0.50, *P* < 0.0001). Similarly, plasma GDF‐15 concentrations were associated with these factors.

**Conclusions:**

Traumatic acid might represent a potential plasma biomarker of sarcopenia. However, further studies are needed to validate the results and investigate the underlying mechanisms.

## Introduction

Sarcopenia, a major modifiable cause of geriatric frailty, is characterized by age‐related involuntary loss of skeletal muscle mass, quality, and strength.^1^ The prevalence of sarcopenia was found to increase from 13–24% in persons younger than 70 years to >50% in persons older than 80 years.^2^ Sarcopenia is associated with poor balance, decreased motility and function, low gait speeds, and a greater risk of falls and fracture.^3^, ^4^ Thus, sarcopenia links poor muscle function to increased all‐cause mortality rates in older people and represents a leading cause of disability, morbidity, and mortality.^5^ Sarcopenia is also associated with higher medical costs. For example, in the USA, the healthcare cost of sarcopenia was estimated to be $18.5bn in 2000.^6^ Accordingly, sarcopenia is a serious issue in geriatrics.

Several possible mechanisms have been suggested for sarcopenia; however, none could fully explain the underlying pathophysiology. Wasting of muscle mass and function is believed to be a complex and multifactorial process, including decreased physical activity, decreased energy intake, poor nutritional status, deteriorated immunity, and metabolic disturbances.^7^ Numerous factors have been identified to be associated with the pathogenesis of sarcopenia, including increased production of inflammatory cytokines (such as interleukin‐6), vitamin D deficiency, insulin resistance, and growth hormone and insulin‐like growth factor‐1 deficiency, low plasma irisin, and elevated plasma growth differentiation factor 15 (GDF‐15).^8^, ^9^, ^10^, ^11^ From a translational perspective, the identification of potential biomarkers represents a promising strategy for exploring the mechanism of sarcopenia.^12^

Metabolomics is an approach to quantitatively measure the dynamic multi‐parametric metabolic responses of living systems to pathophysiological stimuli.^13^ Metabolomics is instrumental for understanding the pathogenesis and diagnosis of diseases^14^ and has been recognized as a powerful tool for assessing complex disease mechanisms, such as systemic lupus erythematosus (SLE).^15^ Searching for potential markers of sarcopenia using the metabolomic approach is thus a reasonable strategy. Serum is often used as an object of metabolomic analysis as the serum metabolome is rich in various classes of important metabolites.^16^, ^17^ Two studies had adopted metabolomic approaches to identify certain plasma amino acids associated with muscle mass and quality in middle‐aged UK female twins^18^ and older people from the Baltimore Longitudinal Study of Aging.^19^ A study also identified some metabolites associated with circulating interleukin‐6 in older adults,^20^ and a recent study revealed the differences in skeletal muscle between nonsarcopenic and sarcopenic older adults using metabolomics.^21^ However, these studies did not compare the differences in plasma metabolites between elderly subjects with and without sarcopenia. As a result, the aim of this study was to identify potential biomarkers of sarcopenia through metabolomic analyses of the plasma metabolites in sarcopenic elderly subjects.

## Methods

### Ethics statement

The study protocol was approved by the Ethics Committee of the National Taiwan University Hospital (Registration Number: 200701017R).^22^ Written informed consent was obtained from all participants before their inclusion in the study. The items included on the consent form were aims, inclusion and exclusion criteria, procedures, potential harm and benefit, medical care received, privacy and right of the participants, and the right to withdraw. All procedures were in accordance with the Declaration of Helsinki. Further, subjects that declined to participate or otherwise did not participate were assured that they would remain in the care of their family physician and would not be subjects to any disadvantages.

### Subjects

Elderly subjects in this study were selected according to the criteria for sarcopenia from the Comprehensive Geriatric Assessment and Frailty Study of Elderly Outpatients.^22^, ^23^ All geriatric ambulatory outpatients (with chronic diseases) were eligible for recruitment if they had one of the following conditions: (i) functional decline (as measured by new disabilities of activity of daily living or instrumental activity daily living), (ii) geriatric syndromes (fall, weight loss, multiple co‐morbidities, etc.), (iii) behavioural disorders (depression or dementia), (iv) expected high healthcare utilization, or (v) age 80 and older. Subjects who were bedridden, resided in nursing homes for long‐term conditions, had a life expectancy of <6 months, and had impaired vision, hearing, or communication capacity were excluded.

All elderly participants were subjected to body composition examination by bioelectrical impedance analysis.^23^, ^24^, ^25^ In accordance with the characteristics of this bioelectrical impedance analysis model (Tanita BC‐418, Tanita Corp., Tokyo, Japan), a constant high‐frequency current (50 kHz, 500 μA) and an eight‐contact electrode were employed to measure the body composition in segmental parts of the whole body, including both arms, legs, and the trunk area. The subjects dressed in light clothing, in a fasted state, and after voiding, were asked to stand on the analyser barefooted in close contact with the electrodes and grasp both hand holders as shown in the user's manual. Fat mass, fat‐free mass, the predicted muscle mass of the appendicular fractions, and appendicular skeletal muscle mass (ASM) could be estimated by the sum of each segment, except for the ‘trunk part’, as validated previously.^25^ Of note, the appendicular skeletal muscle mass index (ASMI) could be estimated via this model (ASMI = ASM divided by squared height in metres). All examinations were conducted in compliance with the standard procedure.^23^, ^24^ Subjects on medical devices were excluded for safety concerns. Elderly subjects were divided according to the presence or absence of sarcopenia using the criteria of low muscle mass (narrow definition of sarcopenia) based on the norm of domestic young healthy adults.^23^, ^26^ The cut‐off points of sarcopenia are 6.76 kg/m^2^ for men and 5.28 kg/m^2^ for women, as validated in previous studies.^23^, ^25^ Additionally, sex‐matched and age‐matched (within 5 year interval) elderly subjects without sarcopenia were selected as the control group. We randomly recruited the same number of adults who were younger than 65, consecutively received annual physical examinations at the outpatient clinics in the same hospital, and did not have malignancies, acute or chronic infections, to serve as another control group.

### Data collection

Data were collected by experienced nurses using a structured questionnaire with the following: demographic information, diseases, smoking and drinking habits, current medication, geriatric syndromes, blood pressure level, and body mass index (BMI).^22^ Body weight and standing height were measured with subjects dressed in light clothing and barefooted.

### Biochemical assays

Blood samples were obtained from the antecubital vein after an 8 h fast to measure complete blood count and biochemical analysis. Blood was immediately centrifuged to obtain plasma samples, which were subsequently frozen at −80°C until analysis. Plasma tumour necrosis factor‐α (TNF‐α) levels were measured using commercial enzyme‐linked immunosorbent assay (ELISA) kits (Assaypro LLC, Saint Charles, MO, USA); the intraassay and interassay coefficients of variation were 5.6% and 7.5%, respectively. Plasma C‐reactive protein (CRP) levels were measured using the latex agglutination test (Denka Seiken, Gosen, Niigata, Japan); the intraassay and interassay coefficients of variation were 4.0% and 8.5%, respectively. Plasma irisin levels were measured using commercial ELISA kits (Cell Biolabs, San Diego, CA, USA); the intraassay and interassay coefficients of variation were 7.8% and 6.2%, respectively. Plasma GDF‐15 levels were measured using commercial ELISA kits (BioVendor, Karasek, Brno, Czechia); the intraassay and interassay coefficients of variation were 2.0% and 7.9%, respectively. All samples were measured according to the manufacturer's recommended procedures and were tested in duplicate.

### Experimental method for metabolic analysis of plasma samples

#### Chemicals

Mass spectrometry (MS)‐grade water and methanol were purchased from Scharlau (Sentmenat, Spain). Acetonitrile was procured from J.T. Baker (Phillipsburg, NJ, USA). Formic acid of 99% concentration was obtained from Sigma‐Aldrich (St. Louis, MO, USA).

#### Sample preparation

Plasma samples were stored at −80°C before use. Before extraction, the samples were thawed at room temperature. Four hundred microlitres of methanol was added to 100 μL of human plasma to extract metabolites from plasma. The extraction was performed using Geno/Grinder2010 (SPEX, Metuchen, NJ, USA) at 1000 r.p.m. for 2 min. Thereafter, the samples were centrifuged at 15 000 *g* for 5 min at 4°C. The extraction was repeated twice. Four hundred microlitres of the supernatant was collected and dried under nitrogen stream. For liquid chromatography (LC)–MS profiling, the dried extracts were reconstituted with 200 μL of 50% methanol and centrifuged at 15 000 *g* for 5 min. The supernatant was then filtered with a 0.2 μm Minisart RC4 filter (Sartorius Stedim Biotech GmbH, Göttingen, Germany). All aliquots were transferred to a glass insert for LC–MS analysis.

#### Metabolomic profiling

Metabolomic profiling via LC–MS was performed using the Agilent 1290 UHPLC system (Agilent Technologies, Santa Clara, CA, USA) coupled with Bruker maXis QTOF (Bruker Daltonics, Bremen, Germany). A 2 μL sample was injected into an Acquity HSS T3 column (2.1 × 100 mm, 1.8 μm) (Waters, Milford, MA, USA) maintained at 40°C. The mobile phase was composed of solvent A: water/0.1% formic acid and solvent B: acetonitrile/0.1% formic acid. The following gradient elution programme was employed: 0–1.5 min: 2% B; 1.5–9 min: linear gradient from 2% to 50% B; and 9–14 min: linear gradient from 50% to 95% B, and maintained at 95% B for 3 min. The flow rate was 300 μL/min. For sample ionization, an electrospray ionization source was employed with a capillary and endplate offset voltage of 4 K and 500 V, respectively, in both positive and negative modes. The MS parameters were set as follows: 200°C, drying gas temperature; 8 L/min, drying gas flow; and 2 bar, nebulizer flow. The mass spectrometer was calibrated with 5 mM sodium formate before daily use with lockmass between runs.

#### Plasma sample preparation for traumatic acid quantification

A 100 μL volume of plasma sample was extracted with 400 μL methanol. The extraction was performed via shaking at 1000 r.p.m. for 2 min using Geno/Grinder 2010 (SPEX SamplePrep). The extract was then centrifuged using the Eppendorf Centrifuge 5810R at 15 000 *g* for 5 min at 4°C. The supernatant was collected in another Eppendorf tube and evaporated using the EYELA CVE‐200D Centrifugal Evaporator (TOKYO RIKAKIKAI CO., Tokyo, Japan) until dry. The residue was re‐reconstituted in 1000 μL of 50% methanol. The reconstituted sample was sonicated for 10 min and centrifuged at 15 000 *g* for 5 min at 4°C. The supernatant was then filtered using a 0.2 μm Minisart RC4 filter (Sartorius Stedim Biotech GmbH) and subjected to LC–MS/MS analysis.

#### Liquid chromatography–tandem mass spectrometry method for traumatic acid quantification

Traumatic acid was analysed using Agilent 1290 UHPLC coupled with an Agilent 6460 triple quadrupole mass spectrometer (Agilent Technologies). The separation was performed on a Phenomenex Kinetex C18 column (2.1 × 50 mm, 2.6 μm, Phenomenex, Torrance, CA, USA), and the column was maintained at 40°C during the analysis. The mobile phase was composed of solvent A (0.1% formic acid in water) and solvent B (0.1% formic acid in acetonitrile). A 0.3 mL/min linear gradient elution was employed as follows: 0–1.5 min, 5% solvent B; 1.5–5 min, 5–95% solvent B; 5–7 min, 95–95% solvent B; and column re‐equilibration with 5% solvent B for 1 min. The injection volume was 5 μL. Negative electrospray ionization mode was utilized with the following parameters: 325°C, drying gas temperature; 8 L/min, drying gas flow; 45 psi nebulizer pressure; 325°C, sheath gas temperature; 11 L/min, sheath gas flow rate; and 3500 V, capillary voltage. Nozzle voltage was set at 500 V. The mass spectrometer was configured in multiple reaction monitoring mode, and the monitored transitions for traumatic acid were *m*/*z* 227.1 → 183.1 and 227.1 → 165. The concentrations of traumatic acid in samples were determined using the peak area of the analyte.

### Data analysis

Mass spectrometry raw files were converted to the mzXML format using Trapper (ISB).^27^ The mzXML data were processed using our in‐house package, TIPick,^28^ which was developed to remove background signals and detect each user‐specific metabolite for UHPLC–MS data. By subtracting the blank chromatogram, TIPick can eliminate chemical signals appearing in blank injections. For target analysis, TIPick utilizes the length and intensity of chromatographic peaks to perform chromatographic peak enhancement and detection. Data analyses were performed using the R statistical software (Version 2.14.2).^29^

### Statistical analyses

The *t*‐tests or Wilcoxon–Mann–Whitney tests, and analyses of variance (ANOVAs) or Kruskal–Wallis tests were used to compare the distribution of continuous variables among elderly subjects without sarcopenia, elderly subjects with sarcopenia, and younger adults. For the categorical variables, *χ* ^2^ tests or Fisher's exact tests were used to assess the difference in proportion between the different groups.

In the screening stage, *t*‐tests or Wilcoxon–Mann–Whitney tests were performed to determine the mean differences of metabolites between elderly subjects without sarcopenia and younger adults, and between elderly subjects with and without sarcopenia, respectively. The metabolites that did not differ between elderly subjects without sarcopenia and young adults and those that significantly differed between elderly subjects with and without sarcopenia were subjected to the analyses mentioned in this study.

Finally, the relationships between metabolites, physical examination, and laboratory tests were evaluated using the Pearson/Spearman correlation coefficients. Statistical analyses were performed using SAS 9.4 (SAS Institute, Cary, NC, USA) and a *P* value <0.05 was considered statistically significant.

## Results

**Figure 1:**
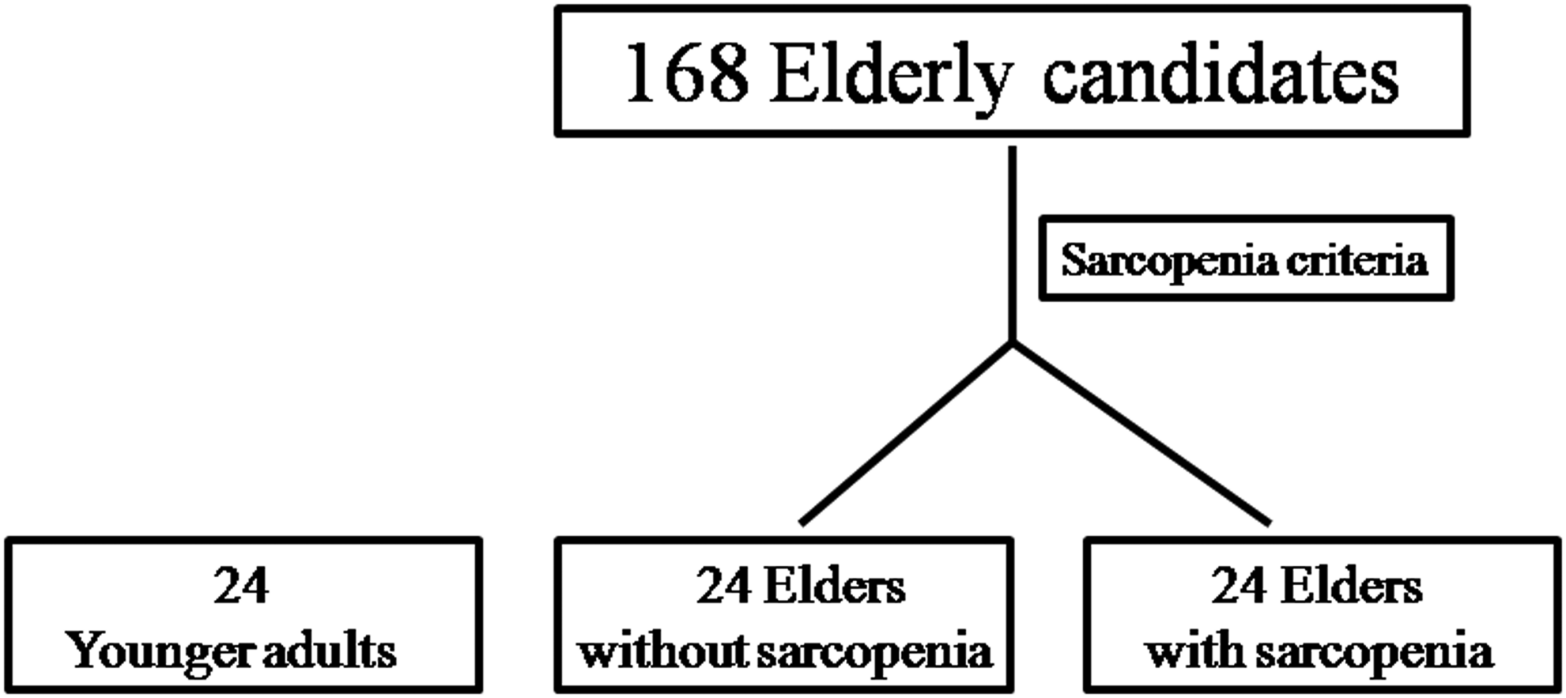
CONSORT diagram of study subjects.

Among the 168 candidates in our previous Comprehensive Geriatric Assessment and Frailty Study of Elderly Outpatients, 24 elderly subjects (≥65 years of age) met the diagnostic criteria for sarcopenia. Additionally, 24 sex‐matched and age‐matched elderly subjects without sarcopenia were employed as the control group. A total of 72 participants were enrolled in the analyses, including 24 elderly subjects without sarcopenia, 24 elderly subjects with sarcopenia, and 24 younger adults [age range: 23–37 years, 12 (50%) men] (*Figure* 1). Demographic data are summarized in *Table* 1. Ten (41.67%) men and 14 (58.33%) women were enrolled in both groups of elderly subjects. The median ages were 82 (range: 67–88) years and 81.5 (range: 67–87) years for the groups of elderly subjects with and without sarcopenia, respectively. There were no significant differences in age, sex distribution, and smoking status between these two groups. The co‐morbidities were not significantly different between elderly subjects with and without sarcopenia, except for hypertension (*P* = 0.0045), and most medications taken by these two groups did not significantly differ, except for beta‐blockers (*P* = 0.033) and calcium channel blockers (*P* = 0.0192).

**Table 1:** Demographic data of study participants

Variable	Elders without sarcopenia (*n* = 24)	Elders with sarcopenia (*n* = 24)	Young adults (*n* = 24)	*P* value (two groups)^a^	*P* value (three groups)^b^
Age (mean ± SD, years)	79.0 ± 5.9	79.4 ± 6.2	29.3 ± 4.3	0.7483^c^	<0.0001^d^
Sex				1.0000	0.7985
Male	10 (41.7)	10 (41.7)	12 (50.0)		
Female	14 (58.3)	14 (58.3)	12 (50.0)		
Smoking status				0.3412^e^	0.0508^e^
Never	15 (62.5)	18 (75.0)	22 (91.7)		
Quitted	9 (37.5)	5 (20.8)	2 (8.3)		
Smoking	0 (0.0)	1 (4.2)	0 (0.00)		
Co‐morbidity					
Hypertension	23 (95.8)	15 (62.5)	0 (0.0)	0.0045	<0.0001
Hyperlipidaemia	12 (50.0)	9 (37.5)	2 (8.3)	0.3827	0.0064
Diabetes mellitus	11 (45.8)	11 (45.8)	0 (0.0)	1.0000	0.0004
Coronary artery disease	8 (33.3)	4 (16.7)	0 (0.0)	0.1824	0.0045^e^
Stroke	8 (33.3)	10 (41.7)	0 (0.0)	0.5510	0.0020
Medication					
Aspirin	11 (45.8)	12 (50.0)	0 (0.0)	0.7726	0.0002
Beta‐blockers	8 (33.3)	2 (8.3)	0 (0.0)	0.0330	0.0026^e^
Calcium channel blockers	14 (58.3)	6 (25.0)	0 (0.0)	0.0192	<0.0001
ACEIs or ARBs	16 (66.7)	12 (50.0)	0 (0.0)	0.2416	<0.0001
Metformin	5 (20.8)	6 (25.0)	0 (0.0)	0.7313	0.0328^e^
Sulfonylureas	7 (29.2)	7 (29.2)	0 (0.0)	1.0000	0.0071^e^
Thiazolidinediones	3 (12.5)	2 (8.3)	0 (0.0)	1.0000^e^	0.3580^e^
Acarbose	0 (0.0)	2 (8.3)	0 (0.0)	0.4894^e^	0.3239^e^
Repaglinide	0 (0.0)	1 (4.2)	0 (0.0)	1.0000^e^	1.0000^e^
Statins	7 (29.2)	8 (33.3)	0 (0.0)	0.7555	0.0082

ACEIs, angiotensin‐converting enzyme inhibitors; ARBs, angiotensin II receptor blockers; SD, standard deviation.

^a^ Elders without sarcopenia vs. elders with sarcopenia.

^b^ Among elders without sarcopenia, elders with sarcopenia, and young adults.

^c^ Wilcoxon–Mann–Whitney test.

^d^ Kruskal–Wallis test.

^e^ Fisher's exact test.

A comparison of the physical examinations and laboratory tests among elderly subjects without sarcopenia, elderly subjects with sarcopenia, and younger adults is summarized in *Table* 2. The medians of weight (64.1 vs. 50.7 kg), BMI (26.3 vs. 21.2 kg/m^2^), ASMI (6.5 vs. 5.3 kg/m^2^), fat mass percentage (37.7% vs. 31.7%), waist circumference (94.5 vs. 82.0 cm), muscle strength (21.0 vs. 15.0 kg), and gait speed (0.9 vs. 0.7 m/s) of elderly subjects without sarcopenia were significantly higher than those of elderly subjects with sarcopenia. However, in the biochemical analyses, except for triglyceride (1.7 vs. 1.3 mmol/L, *P* = 0.0370), there was no significant difference between elderly subjects without sarcopenia and elder subjects with sarcopenia in red blood cell, haemoglobin, platelet, white blood cell, neutrophil, lymphocyte, albumin, glucose AC, total cholesterol, aspartate aminotransferase, alanine aminotransferase, blood urine nitrogen, creatinine, uric acid, log‐transformed median plasma TNF‐α (pg/mL), log‐transformed median plasma CRP (nmol/mL), log‐transformed median plasma irisin (ng/mL), and log‐transformed median plasma GDF‐15 (pg/mL).

**Table 2:** Results of the physical examination and laboratory tests

Variable	Elders without sarcopenia (*n* = 24)		Elders with sarcopenia (*n* = 24)		Young adults (*n* = 24)		*P* value^a^ (two groups)	*P* value^b^ (three groups)
	Median	(Q1, Q3)	Median	(Q1, Q3)	Median	(Q1, Q3)		
Physical examination								
Height (cm)	155.1	(149.5, 161.8)	155.2	(150.8, 163.6)	165.6	(162.8, 175.3)	0.6467	<0.0001
Weight (kg)	64.1	(58.1, 69.7)	50.7	(46.4, 55.2)	62.3	(54.1, 75.5)	<0.0001	<0.0001
Body mass index (kg/m^2^)	26.3	(25.1, 27.0)	21.2	(19.7, 22.0)	22.5	(20.9, 25.4)	<0.0001	<0.0001
ASMI (kg/m^2^)	6.5	(5.8, 7.7)	5.3	(5.1, 6.3)	NA		<0.0001^d^	
Fat mass percentage (%)	37.7	(30.7, 43.1)	31.7	(22.3, 35.9)	NA		0.0080	
Waist circumstance (cm)	94.5	(88.0, 98.5)	82.0	(77.8, 85.5)	79.0	(70.8, 87.0)	<0.0001	<0.0001
Muscle strength (kg)	21.0	(16.0, 31.0)	15.0	(12.0, 21.5)	NA		0.0231	
Gait speed (m/s)	0.9	(0.6, 1.3)	0.7	(0.5, 0.9)	NA		0.0461	
Blood pressure								
Systolic (mmHg)	129.0	(119.0, 139.0)	130.0	(119.5, 138.5)	112.0	(108.5, 121.5)	0.9433	0.0005
Diastolic (mmHg)	70.0	(69.0, 76.0)	71.5	(69.5, 80.0)	78.0	(67.5, 83.5)	0.2880^d^	0.2010^e^
Laboratory tests								
RBC (M/μL)	4.4	(3.8, 4.5)	4.3	(4.1, 4.5)	4.8	(4.4, 5.3)	0.5983	0.0004^e^
Haemoglobin (g/dL)	12.7	(11.3, 13.3)	13.0	(12.4, 13.7)	13.9	(13.1, 15.4)	0.3217	0.0014^e^
Platelet (K/μL)	240.5	(189.5, 272.5)	223.0	(185.0, 265.0)	259.5	(227.5, 289.5)	0.7571^d^	0.0989^e^
WBC (K/μL)	6.2	(5.5, 7.2)	6.5	(5.5, 7.0)	6.5	(6.1, 7.8)	0.8874	0.2924
Neutrophil (%)	57.0	(53.5, 62.4)	60.5	(49.9, 66.3)	59.1	(56.1, 63.2)	0.6678	0.7083
Lymphocyte (%)	33.6	(28.7, 35.7)	30.5	(25.6, 42.8)	32.0	(30.0, 35.2)	0.8778	0.8619
Albumin (g/dL)	4.5	(4.4, 4.0)	4.6	(4.4, 4.8)	4.9	(4.9, 5.1)	0.5829	0.0003
Glucose AC (mmol/L)	5.7	(5.2, 7.4)	5.8	(4.9, 7.4)	4.6	(4.3, 4.9)	0.7334^d^	<0.0001^e^
Total cholesterol (mmol/L)	5.0	(4.4, 5.8)	4.6	(4.1, 5.2)	4.9	(4.5, 5.5)	0.2374^d^	0.4305^e^
Triglyceride (mmol/L)	1.7	(1.1, 2.4)	1.3	(0.8, 1.9)	0.9	(0.6, 1.2)	0.0370^d^	0.0007^e^
AST (μkat/L)	0.3	(0.3, 0.4)	0.4	(0.3, 0.9)	0.3	(0.3, 0.4)	0.1291^d^	0.2931^e^
ALT (μkat/L)	0.3	(0.2, 0.4)	0.3	(0.2, 0.4)	0.3	(0.2, 0.5)	0.4955^d^	0.1559^e^
BUN (mmol/L)	6.0	(4.8, 9.4)	6.8	(4.7, 8.3)	3.8	(3.2, 4.6)	0.9124^d^	<0.0001^e^
Creatinine (μmol/L)	88.4	(84.0, 110.5)	97.2	(79.6, 123.8)	84.0	(70.7, 97.2)	0.9834^d^	0.0802^e^
MDRD‐simplify‐GFR^c^ (mL/min/1.73 m^2^)	57.4	(46.2, 74.4)	59.3	(46.7, 71.4)	88.5	(83.3, 92.8)	0.9151	<0.0001
Uric acid (μmol/L)	362.9	(321.2, 386.7)	336.1	(276.6, 419.4)	315.3	(291.5, 386.7)	0.3967^d^	0.6120^e^
Log (TNF‐α (pg/mL))	1.6	(1.5, 1.7)	1.6	(1.2, 1.8)	NA		0.6567^d^	
Log (CRP (nmol/L))	1.3	(1.2, 1.8)	1.3	(1.1, 1.5)	NA		0.1801^d^	
Log (irisin (ng/mL))	2.8	(2.5, 3.2)	3.1	(2.2, 3.3)	2.4	(2.0, 3.0)	0.2376^d^	0.0427^e^
Log (GDF‐15 (pg/mL))	6.1	(5.7, 6.5)	6.2	(5.6, 6.6)	4.8	(4.6, 5.0)	0.5728^d^	<0.0001^e^

ALT, alanine aminotransferase; ASMI, appendicular skeletal muscle index; AST, aspartate aminotransferase; BUN, blood urine nitrogen; CRP, C‐reactive protein; GDF‐15, growth differentiation factor 15; GFR, glomerular filtration rate; MDRD, Modification of Diet in Renal Disease; RBC, red blood cell; TNF‐α, tumour necrosis factor‐α; WBC, white blood cell.

^a^ Elders without sarcopenia vs. elders with sarcopenia.

^b^ Among elders without sarcopenia, elders with sarcopenia, and young adults.

^c^ MDRD‐simplify‐GFR (mL/min/1.73 m^2^) = 186 × [(CRE)^−1.154^] × [(age)^−0.203^] (if male); MDRD‐simplify‐GFR (mL/min/1.73 m^2^) = 186 × [(CRE)^−1.154^] × [(age)^−0.203^] × 0.742 (if female).

^d^ Wilcoxon–Mann–Whitney test.

^e^ Kruskal–Wallis test.

In the metabolomics analyses, a total of 242 metabolic peaks were detected, annotated, and listed in Supporting Information, *Table* S1. To visualize the intensity level of each detected metabolic peak, the intensity levels of metabolic peaks were natural log‐transformed and the log‐transformed levels of detected metabolic peaks were encoded into heatmap representations (*Figure* S1). The pathway analysis integrated in MetaboAnalyst with default parameters was used to analyse the detected metabolites.^30^ Based on pathway analysis, 13 pathways were covered by the detected metabolites, with a *P* value <0.05 (*Table* S2).

**Figure 2:**
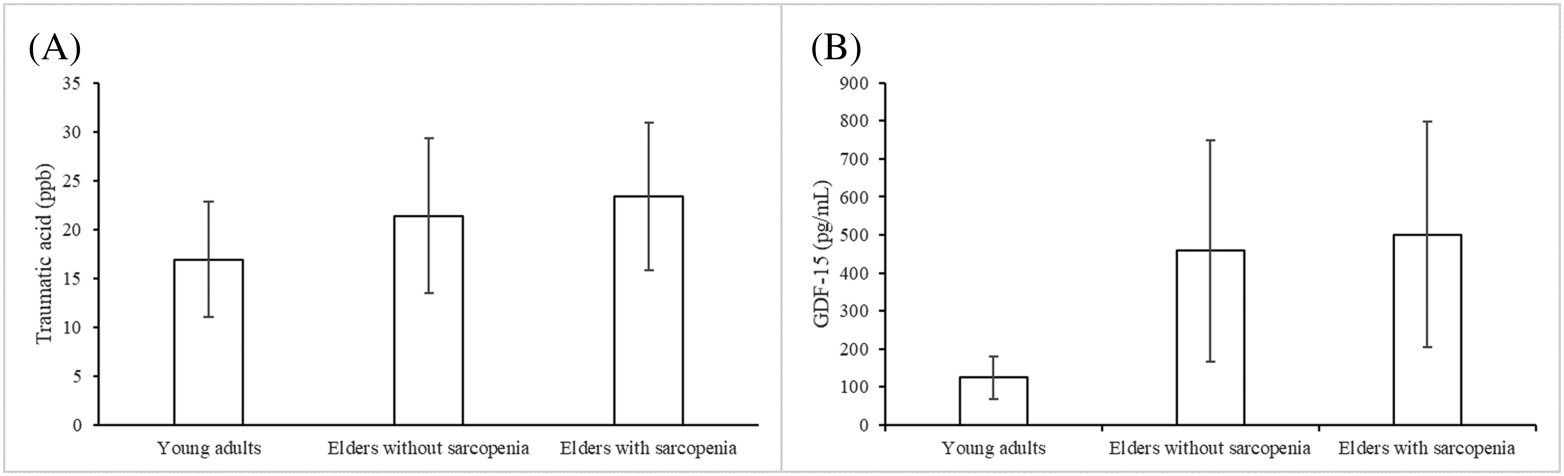
Plasma (A) traumatic acid and (B) growth differentiation factor 15 (GDF‐15) concentrations in younger adults, elderly subjects without sarcopenia, and elderly subjects with sarcopenia. The *P* value for testing the linear trend using analysis of variance was 0.034 and <0.001 for traumatic acid and GDF‐15, respectively. Based on *post hoc* analyses, there was no difference in the concentration level between elderly subjects with and without sarcopenia for traumatic acid (*P* = 0.61) and GDF‐15 (*P* = 0.812). The levels in young adults were lower than those in elderly people without sarcopenia (*P* = 0.087) and with sarcopenia (*P* = 0.008) for traumatic acid as well as for GDF‐15 (*P* < 0.001 and *P* < 0.001, respectively).

In the screening stage, Wilcoxon–Mann–Whitney test was performed to compare elderly subjects without sarcopenia and younger adults to identify ageing‐associated metabolites. Subsequently, the same analysis between elderly subjects without sarcopenia and elderly subjects with sarcopenia was conducted to identify ageing and/or sarcopenia‐associated metabolites. The level of significance was corrected as 0.025 for multiple comparisons using Bonferroni correction. According to the selection criteria, these statistical analyses suggest that traumatic acid might be considered a sarcopenia‐related metabolite. The plasma traumatic acid intensity level of elderly subjects with sarcopenia was significantly higher than that of elderly subjects without sarcopenia (591.5 vs. 430.0, *P* = 0.0063). Linear regression (*R* ^2^ = 0.23) was further performed to determine the association between traumatic acid and sarcopenia (with vs. without, *β* = 249.57, *P* = 0.01) after controlling for hypertension status (yes vs. no, *β* = −55.48, *P* = 0.63), glucose AC (*β* = −37.32, *P* = 0.15), and creatinine (*β* = 0.70, *P* = 0.63). However, no difference in the plasma intensity level of traumatic acid was found between elderly subjects without sarcopenia and younger adults (*Table* 3). The plasma concentrations of traumatic acid in the three groups were further determined using the LC–MS/MS method. Based on the results, the concentrations were 15.8, 21.1, and 24.3 ppb in younger adults, elderly subjects without sarcopenia, and elderly subjects with sarcopenia, respectively (*Table* 3). Although the data obtained from LC–MS/MS did not validate the data from LC–QTOF analysis, we assessed both datasets from the two platforms that revealed similar trends with positive correlation (Pearson's correlation: *r* = 0.25, *P* = 0.0325). The difference and linear trend of plasma traumatic acid concentrations in three groups were also explored using ANOVA (*Figure* 2A). Accordingly, a significant linear trend (*P* = 0.034) was found in the three groups. Although no difference was found in the concentration between elderly subjects with and without sarcopenia (*P* = 0.61), the concentration of plasma traumatic acid for young adults was lower than that for elderly subjects without sarcopenia (*P* = 0.087) and with sarcopenia (*P* = 0.008) based on *post hoc* analyses. The pattern of traumatic acid was similar to that of GDF‐15, a recognized marker for sarcopenia (*P* for trend <0.001) (*Figure* 2B).

**Table 3:** Plasma levels of traumatic acid in different groups

Metabolite	Elders without sarcopenia (*n* = 24)		Elders with sarcopenia (*n* = 24)		Young adults (*n* = 24)		*P* value^a^	*P* value^b^
	Median	(Q1, Q3)	Median	(Q1, Q3)	Median	(Q1, Q3)		
Traumatic acid (signal intensity level)	430.0	(261.0, 599.5)	591.5	(491.5, 664.5)	362.5	(321.0, 454.5)	0.6517	0.0063^c^
Traumatic acid (ppb)	21.1	(16.0, 25.8)	24.3	(18.0, 29.5)	15.8	(11.5, 21.7)	0.0539^c^	0.3274^c^

^a^ Young adults vs. elders without sarcopenia.

^b^ Elders without sarcopenia vs. elders with sarcopenia.

^c^ Wilcoxon–Mann–Whitney test.

Correlation analyses were further performed to investigate the relationship between traumatic acid, GDF‐15, and the parameters of sarcopenia and nutrition status. Both plasma traumatic acid and GDF‐15 concentrations were not associated with ASMI, muscle strength, and gait speed. However, plasma traumatic acid concentrations were positively correlated with the presence of hypertension (*P* = 0.034), glucose AC (*P* = 0.0035), creatinine (*P* = 0.0006), and GDF‐15 levels (*P* = 0.0376), but negatively correlated with the Modification of Diet in Renal Disease‐simplify‐glomerular filtration rate (*P* < 0.0001) (*Table* 4). Similarly, plasma GDF‐15 concentrations were positively correlated with the presence of hypertension (*P* = 0.0017), glucose AC (*P* = 0.0002), and creatinine levels (*P* = 0.0056), but negatively correlated with haemoglobin (*P* = 0.0054), albumin (*P* = 0.0022), and the Modification of Diet in Renal Disease‐simplify‐glomerular filtration rate (*P* < 0.0001) (*Table* 4).

**Table 4:** Correlation between plasma traumatic acid and GDF‐15, physical examination, and laboratory tests

Variable	Traumatic acid (ppb)		GDF‐15^a^	
	Correlation coefficient	*P* value	Correlation coefficient	*P* value
Body mass index (kg/m^2^)	−0.10	0.3860	−0.06	0.6054
ASMI (kg/m^2^)	−0.03^a^	0.8438	−0.06	0.7124
Fat mass percentage (%)	−0.09	0.5259	−0.08	0.6115
Waist circumstance (cm)	0.06	0.5931	0.22	0.0679
Muscle strength (kg)	0.02	0.8668	−0.23	0.1119
Gait speed (m/s)	−0.10	0.5022	−0.11	0.4591
Hypertension (categorical, no: 0; yes: 1)	0.25	0.0340	0.37	0.0017
Haemoglobin (g/dL)	−0.09^a^	0.4377	−0.32	0.0054
NLR	0.11^a^	0.4173	0.11	0.4522
PLR	−0.03^a^	0.8307	−0.08	0.5843
SII	−0.01^a^	0.9520	0.03	0.8255
Albumin (g/dL)	−0.08	0.5697	−0.42	0.0022
Glucose AC (mmol/L)	0.34^a^	0.0035	0.44	0.0002
Total cholesterol (mmol/L)	−0.01^a^	0.9228	−0.05	0.7049
Triglyceride (mmol/L)	0.02^a^	0.8873	0.23	0.0595
ALT (μkat/L)	−0.22^a^	0.0588	−0.13	0.2694
Creatinine (μmol/L)	0.40^a^	0.0006	0.33	0.0056
MDRD‐simplify‐GFR (mL/min/1.73 m^2^)	−0.50	<0.0001	−0.60	<0.0001
Uric acid (μmol/L)	0.07^a^	0.5408	0.19	0.1129
Log (TNF‐α (pg/mL))	−0.05^a^	0.7440	0.12	0.4158
Log (CRP (nmol/L))	0.15^a^	0.2992	−0.12	0.4088
GDF‐15 (pg/mL)	0.25^a^	0.0376		

ALT, alanine aminotransferase; ASMI, appendicular skeletal muscle index; CRP, C‐reactive protein; GDF‐15, growth differentiation factor 15; GFR, glomerular filtration rate; MDRD, Modification of Diet in Renal Disease; NLR, neutrophil/lymphocyte ratio; PLR, platelet/lymphocyte ratio; SII, systemic immune‐inflammation index; TNF‐α, tumour necrosis factor‐α.

^a^ Spearman correlation.

## Discussion

To identify the biomarkers of sarcopenia in this study, we performed metabolomic analyses and demonstrated that plasma traumatic acid intensity levels did not differ between younger adults and elderly subjects without sarcopenia, but were higher in elderly subjects with sarcopenia than those without sarcopenia. For further validation, we assayed the plasma concentrations of traumatic acid in these three groups using the LC–MS/MS method. As the sensitivity between the two platforms was different and the sample size was small, a slight difference may be obtained in the statistical analysis, especially for less abundant metabolites, such as traumatic acid. However, we examined both datasets from two platforms and found that both revealed similar trends with positive correlation. Moreover, plasma traumatic acid concentrations displayed a significant tendency to elevate progressively in three groups, from younger adults to elderly subjects without sarcopenia, and to elderly subjects with sarcopenia. These findings suggest that traumatic acid might be relevant for the development of sarcopenia.

Although there was no difference in plasma TNF‐α and CRP levels in this small‐scale study, the data of our Comprehensive Geriatric Assessment and Frailty Study of Elderly Outpatients revealed higher CRP levels and inflammatory markers, such as intercellular adhesion molecule‐1 and TNF‐α mRNA expression in peripheral blood mononuclear cells of elderly subjects than younger adults.^31^ Several studies also demonstrated that the pathophysiology of sarcopenia is very complex and is associated with chronic inflammation as well as the increased production of pro‐inflammatory cytokines and prostaglandins.^8^, ^32^ Traumatic acid is described as a plant wound hormone and is used as an intermediate in prostaglandin synthesis.^33^ Unsaturated fatty acids, such as prostaglandins, perform an important role in maintaining normal physiological functions, including the regulation of immune responses.^34^ Moreover, prostaglandins, such as prostaglandin E_2_ (PGE2), have been shown to play an important role in regulating skeletal muscle adaptations to ageing.^35^ PGE2 may contribute to higher rates of skeletal muscle proteolysis in ageing humans.^36^ A prior study revealed that PGE2 can induce the transcription of skeletal muscle mass regulators, interleukin‐6, and muscle RING finger‐1, in humans, which is an important mechanism for skeletal muscle proteolysis.^32^ Another study further showed that nutritional intervention with specific medical food significantly increased body weight and improved functional performance status, which was accompanied by significantly reduced serum PGE2 levels, in newly diagnosed oesophageal cancer patients.^37^ These findings seem to support our findings of higher plasma traumatic acid levels in elderly subjects with sarcopenia.

In addition to lower muscle mass, muscle strength, and gait speed, elderly subjects with sarcopenia in the present study had lower BMI, fat percentage, and plasma triglyceride levels, suggesting that sarcopenia is a significant wasting and malnutrition status.^7^ Chronic inflammatory diseases and their complications are associated with wasting and malnutrition in sarcopenia. Previously, traumatic acid was suggested to be a differential metabolite involved in the pathogenesis of SLE, a complex systemic autoimmune disease with wasting process; this is because elevated serum levels of traumatic acid were detected in experimented animals with SLE.^15^ A recent study also demonstrated that compared with that in control subjects, plasma traumatic acid levels were higher in patients with inflammatory bowel disease, a chronic inflammatory course of lower digestive tract.^38^ These data also support our data regarding the increasing tendency of plasma traumatic acid levels in elderly subjects with sarcopenia, a potential chronic inflammatory status.

In this study, we found that plasma traumatic acid levels were associated with the presence of hypertension, higher preprandial plasma glucose and GDF‐15 levels, and impaired renal functions. Hypertension and hyperglycaemia are well known as important components of metabolic syndrome. Numerous studies have demonstrated that metabolic syndrome is associated with skeletal muscle abnormalities, including changes in skeletal muscle fibre composition, metabolism, insulin sensitivity, mitochondrial functions, and strength.^39^ Thus, metabolic syndrome and insulin resistance are important factors associated with sarcopenia.^8^, ^39^ Such results might support our tentative finding that traumatic acid is associated with these risk factors for sarcopenia. Traumatic acid concentrations were not found to be associated with ASMI, muscle strength, and gait speed. Such finding may be due to the older participants having chronic diseases and taking several medications, which may act as confounding factors to the study of sarcopenia. Therefore, in the future, the association between traumatic acid and muscle loss would be better assessed in healthier individuals to avoid these confounding effects.

Growth differentiation factor 15 is associated not only with decreased muscle performance, increased inflammation, anaemia, impaired renal functions, and metabolic disorders but also with age‐related sarcopenia.^10^, ^11^ Similarly, we demonstrated that plasma GDF‐15 levels are associated with the presence of hypertension, higher preprandial plasma glucose levels, lower haemoglobin and albumin levels, and impaired renal functions. The concentrations of GDF‐15, similar to those of traumatic acid, did not differ between elderly subjects without sarcopenia and elderly subjects with sarcopenia and were not associated with sarcopenia‐related variables, such as ASMI, muscle strength, and gait speed. A recent study with older Asian adults also reported similar results. These results may be because these patients often suffer from a variety of chronic diseases and the medication for these diseases may affect plasma GDF‐15 concentrations.^40^ However, the tendency is similar as both traumatic acid and GDF‐15 levels progressively elevated in the three groups from younger adults to elderly subjects without sarcopenia, and to elderly subjects with sarcopenia. Interestingly, traumatic acid levels were positively correlated with GDF‐15 levels in plasma. Such findings suggest that traumatic acid, similar to GDF‐15, might be a potential marker for sarcopenia.

To select sarcopenia‐specific markers rather than age‐related markers in our research design for this study, we recruited a group of younger adults for comparison. Metabolites that differ between younger adults and elderly subjects without sarcopenia might be age related and therefore were excluded in the analyses. If other statistical methods, such as discriminant analysis in stepwise methods,^41^ were employed, age‐related markers may be obtained. Therefore, traumatic acid, whose intensity levels differed between elderly subjects without and with sarcopenia, was selected for the analyses.

This study had several limitations. First, the sample size was relatively small. Larger‐scale studies are needed to confirm and refine the results obtained in this study. Second, as this was a cross‐sectional observational study, our findings could not establish causal relationships between plasma traumatic acid levels and sarcopenia. Third, the mean age of study participants was over 80 years; these patients exhibited both muscle and fat wasting, an extremely critical condition. Elderly patients between ages 65 and 80 should be recruited in future studies to track the course and process of sarcopenia.

In conclusion, a metabolomics‐based approach was demonstrated to be a promising strategy for identifying biomarkers of sarcopenia. Owing to this approach, plasma traumatic acid was identified as a potential biomarker of sarcopenia. Further studies are needed to validate the results and clarify the causal relationships of traumatic acid with sarcopenia and the underlying mechanisms.

## Conflict of interest

None declared.

## Funding

This study was supported by grants (NSC 98‐2314‐B‐002‐118‐MY2, MOST 107‐2314‐B‐002‐273, MOST 108‐2314‐B‐002‐106, and MOST 109‐2314‐B‐002‐165‐MY3 to Jaw‐Shiun Tsai; PH‐098‐PP‐48 to Ching‐Yu Chen) from the National Science Council and the National Health Research Institutes, Taiwan. The financial sponsors played no role in any aspect of the study.

## Supporting information


**Table S1.** Annotations of detected metabolic peaks.
**Table S2.** Pathways covered by the detected metabolites.
**Figure S1.** Levels of detected metabolic peaks.
