# Supplementary material for: Identification of traumatic acid as a potential plasma biomarker for sarcopenia using a metabolomics‐based approach
Source: J Cachexia Sarcopenia Muscle. 2021 Dec 22;13(1):276–86. doi: 10.1002/jcsm.12895 (PMC8818620; doi:10.1002/jcsm.12895)
Supplement: Supplementary file 1 — Table S1. Annotations of detected metabolic peaks. Table S2. Pathways covered by the detected metabolites. Figure S1. Levels of detected metabolic peaks. [file JCSM-13-276-s001.docx]

**Table S-1.** Annotations of detected metabolic peaks.

| **Positive** | | | |
| --- | --- | --- | --- |
| **No.** | **Peak annotation** | **No.** | **Peak annotation** |
| 1 | Imidazoleacetic acid | 39 | Isomer of 1-Methylhistidine |
| 2 | Histamine | 40 | Cytosine |
| 3 | Carnosine | 41 | Pipecolic acid |
| 4 | L-Ornithine | 42 | 3-Aminoisobutanoate |
| 5 | D-Lysine | 43 | Isomer of L-Acetylcarnitine |
| 6 | Spermine | 44 | IMP |
| 7 | 1-Methylhistamine | 45 | Isomer of N-Acetylserine |
| 8 | Isomer of Carnosine | 46 | Isomer of Glycylproline |
| 9 | 3-Methylhistidine | 47 | Glutathione |
| 10 | Arginine | 48 | 4-Guanidinobutanoic acid |
| 11 | Isomer of Histamine | 49 | Isomer of N6-Acetyl-L-lysine |
| 12 | Isomer of L-Ornithine | 50 | Isomer of Pipecolic acid |
| 13 | Phosphoserine | 51 | L-Acetylcarnitine |
| 14 | Carnitine | 52 | AMP |
| 15 | Glycerophosphocholine | 53 | Dihydrouracil |
| 16 | D-Glutamine | 54 | Pyroglutamic acid |
| 17 | Serine | 55 | Isomer of IMP |
| 18 | Trimethylamine N-oxide | 56 | Methionine |
| 19 | Taurine | 57 | Normetanephrine |
| 20 | Alpha-Lactose | 58 | Dopamine |
| 21 | N-Acetylserine | 59 | 1-Methyladenosine |
| 22 | Quinone | 60 | Hypoxanthine |
| 23 | Betaine \| L-Valine^*^ | 61 | Isomer of Methionine |
| 24 | Creatinine | 62 | Isomer of Pyroglutamic acid |
| 25 | Allantoin | 63 | Isomer of AMP |
| 26 | L-Alanine \| beta-alanine^*^ | 64 | 5-Methylcytidine |
| 27 | L-Citrulline | 65 | Cotinine |
| 28 | 1-Methylhistidine | 66 | S-Adenosyl-L-homocysteine |
| 29 | Cysteine-S-sulfate | 67 | Isomer of S-Adenosyl-L-homocysteine |
| 30 | 3-Pyridylacetic acid | 68 | 5,6-Dihydrothymine |
| 31 | Isomer of Carnosine | 69 | L-Alloisoleucine |
| 32 | Beta-N-Acetylglucosamine | 70 | Isomer of 5,6-Dihydrothymine |
| 33 | Glycylproline | 71 | Isomer of 5,6-Dihydrothymine |
| 34 | L-Threonine | 72 | L-Norleucine |
| 35 | 2'-Deoxyguanosine 5'-monophosphate | 73 | Adenosine |
| 36 | L-a-aminobutyric acid | 74 | Inosine |
| 37 | N6-Acetyl-L-lysine | 75 | Isomer of Inosine |
| 38 | 5-Aminolevulinic acid | 76 | Monoethyl malonic acid |
| **No.** | **Peak annotation** | **No.** | **Peak annotation** |
| 77 | o-Tyrosine P12-17 | 100 | Ofloxacin |
| 78 | 5-Hydroxy-L-tryptophan | 101 | Hexanoylcarnitine |
| 79 | Adenosine 2',3'-cyclic phosphate | 102 | 3,4,5-Trimethoxycinnamic acid |
| 80 | Deoxyadenosine | 103 | 3,3'5-Triiodo-L-thyronine |
| 81 | Isomer of o-Tyrosine P12-17 | 104 | 3-Indolepropionic acid |
| 82 | L-Kynurenine | 105 | 3-Indolepropionic acid |
| 83 | Isomer of 5-Hydroxy-L-tryptophan | 106 | Corticosterone |
| 84 | 3-Nitrotyrosine | 107 | Isomer of Corticosterone |
| 85 | Glycyl-L-leucine | 108 | Cortexolone |
| 86 | 1,3-Dimethyluracil | 109 | Glycocholate |
| 87 | Isomer of 3-Nitrotyrosine | 110 | Methyl indole-3-acetate |
| 88 | 5'-Methylthioadenosine | 111 | Glycochenodeoxycholate |
| 89 | L-Tryptophan | 112 | Androstenedione |
| 90 | g-Glutamylleucine | 113 | Isomer of Glycochenodeoxycholate |
| 91 | 5-Methoxytryptophan | 114 | Isomer of Androstenedione |
| 92 | Kynurenate | 115 | Androsterone |
| 93 | Tryptamine | 116 | Epitestosterone |
| 94 | Quinaldic acid | 117 | Isomer of Androstenedione |
| 95 | N-Acetylserotonin | 118 | 16-Dehydroprogesterone |
| 96 | 2-Phenylaminoadenosine | 119 | Glycyrrhetinic acid |
| 97 | Biotin | 120 | Isomer of L-Citrulline |
| 98 | Hippurate | 121 | Isomer of L-Acetylcarnitine |
| 99 | D-Pantethine |  |  |
| **Negative** | | | |
| **No.** | **Peak annotation** | **No.** | **Peak annotation** |
| 122 | Mannitol \| D-Sorbitol \| Dulcitol^*^ | 136 | Ribonolactone |
| 123 | D-Ribose | 137 | D-Fructose |
| 124 | Allose | 138 | Dehydroascorbic acid |
| 125 | Isomer of Orotidine | 139 | Malic Acid |
| 126 | Glycerol 3-phosphate | 140 | Orotidine |
| 127 | Galacturonic acid | 141 | Glutaconic acid |
| 128 | Glucose 6-phosphate \|  D-Fructose 6-phosphate^*^ | 142 | D-Glucurono-6,3-lactone |
| 129 | D-Ribose 5-phosphate | 143 | Isomer of 2-Oxoglutarate |
| 130 | Tartarate | 144 | Ascorbic Acid |
| 131 | 2-Oxoglutarate | 145 | Orotic acid |
| 132 | DL-Arabinose | 146 | Pyrrolidonecarboxylic acid |
| 133 | Succinic acid semialdehyde | 147 | Isomer of 2-Oxoglutarate |
| 134 | L-Gulono-1,4-lactone | 148 | Deoxyribose |
| 135 | Pyruvic acid | 149 | citric acid |
| **No.** | **Peak annotation** | **No.** | **Peak annotation** |
| 150 | cis-Aconitate | 189 | 3,4-Dihydroxyhydrocinnamic acid |
| 151 | 2-Oxobutanoate | 190 | 2-Ethyl-2-Hydroxybutyric acid |
| 152 | Isomer of Pyrrolidonecarboxylic acid | 191 | Bradykinin |
| 153 | Uridine | 192 | 2-Ketohexanoic acid |
| 154 | Isomer of Uridine | 193 | Phenylpyruvate |
| 155 | Fumarate | 194 | 2-Hydroxycaproic acid |
| 156 | Acetoacetic acid | 195 | Ortho-Hydroxyphenylacetic acid |
| 157 | Isomer of cis-Aconitate | 196 | N-Acetylleucine |
| 158 | Succinate | 197 | P-Hydroxybenzaldehyde |
| 159 | trans-Aconitic acid | 198 | Isomer of 2-Hydroxycaproic acid |
| 160 | (R)-3-Hydroxybutyric acid \|  (S)-3-Hydroxybutyric acid^*^ | 199 | Isomer of 2-Hydroxycaproic acid |
| 161 | 2-Hydroxybutyric acid | 200 | Isomer of N-Acetylleucine |
| 162 | Isomer of trans-Aconitic acid | 201 | Isomer of Ortho-Hydroxyphenylacetic acid |
| 163 | 3-Hydroxymethylglutaric acid | 202 | Desaminotyrosine |
| 164 | Isomer of 2-Hydroxybutyric acid | 203 | Suberic acid |
| 165 | Adipic acid | 204 | Salicyluric acid |
| 166 | Levulinic acid | 205 | trans-Cinnamic acid |
| 167 | Isomer of Levulinic acid | 206 | 4-Methoxyphenylacetic acid |
| 168 | 3-Hydroxyisovaleric acid | 207 | Phosphorylcholine |
| 169 | Glutaric acid | 208 | Homoveratric acid |
| 170 | 3-Methoxy-4-hydroxymandelate | 209 | Indolelactic acid |
| 171 | Itaconic acid | 210 | Azelaic acid |
| 172 | Isomer of 3-Hydroxyisovaleric acid | 211 | Isomer of Azelaic acid |
| 173 | 3-Furoic acid | 212 | Sebacic acid |
| 174 | Isomer of Itaconic acid | 213 | Isomer of Sebacic acid |
| 175 | 2-Furoylglycine | 214 | Isomer of Sebacic acid |
| 176 | 2-Hydroxy-2-methylbutyric acid | 215 | Isomer of Sebacic acid |
| 177 | Ethylmalonic acid | 216 | Isomer of Hydrocinnamic acid |
| 178 | 4-Hydroxyphenylacetate | 217 | Hydrocinnamic acid |
| 179 | Hydroxyisocaproic acid | 218 | 3-Methylphenylacetic acid |
| 180 | N-Formyl-L-methionine | 219 | Hydroxyoctanoic acid |
| 181 | Isomer of Hydroxyisocaproic acid | 220 | m-Chlorobenzoic acid |
| 182 | Hydroxyphenyllactic acid | 221 | Traumatic acid |
| 183 | 1,3,7-Trimethyluric acid | 222 | Isomer of Traumatic acid |
| 184 | Catechol | 223 | Prostaglandin E2 |
| 185 | Gamma-Caprolactone | 224 | Dodecanedioic acid |
| 186 | 2,2-Dimethylsuccinic acid | 225 | Prostaglandin E1 |
| 187 | Ketoleucine | 226 | Isomer of Dodecanedioic acid |
| 188 | Pimelic acid | 227 | Prostaglandin D2 |
| **No.** | **Peak annotation** | **No.** | **Peak annotation** |
| 228 | 12-Hydroxydodecanoic acid | 236 | Isomer of Hyodeoxycholic acid P6-24 |
| 229 | 1,11-Undecanedicarboxylic acid | 237 | Isomer of Tetradecanedioic acid |
| 230 | Cholic Acid | 238 | Deoxycholic acid |
| 231 | Isomer of 1,11-Undecanedicarboxylic acid | 239 | Isomer of Deoxycholic acid |
| 232 | Isomer of Cholic Acid | 240 | Isomer of Deoxycholic acid |
| 233 | Hyodeoxycholic acid P6-24 | 241 | 5-Dodecenoic acid |
| 234 | Isomer of Cholic Acid | 242 | Lithocholic acid |
| 235 | Tetradecanedioic acid |  |  |

*: A | B denotes that A or B.

**Table S-2.** Pathways covered by the detected metabolites.

| Pathway | Total | Hits | *p* value |
| --- | --- | --- | --- |
| Cysteine and methionine metabolism | 56 | 11 | 0.0004 |
| Citrate cycle (TCA cycle) | 20 | 6 | 0.0009 |
| Phenylalanine metabolism | 45 | 9 | 0.0012 |
| Alanine, aspartate and glutamate metabolism | 24 | 6 | 0.0025 |
| Tyrosine metabolism | 76 | 11 | 0.0055 |
| Butanoate metabolism | 40 | 7 | 0.0093 |
| Propanoate metabolism | 35 | 6 | 0.0174 |
| Glycine, serine and threonine metabolism | 48 | 7 | 0.0244 |
| D-Glutamine and D-glutamate metabolism | 11 | 3 | 0.0256 |
| Pyrimidine metabolism | 60 | 8 | 0.0272 |
| Glyoxylate and dicarboxylate metabolism | 50 | 7 | 0.0299 |
| Synthesis and degradation of ketone bodies | 6 | 2 | 0.0472 |
| Histidine metabolism | 44 | 6 | 0.0483 |


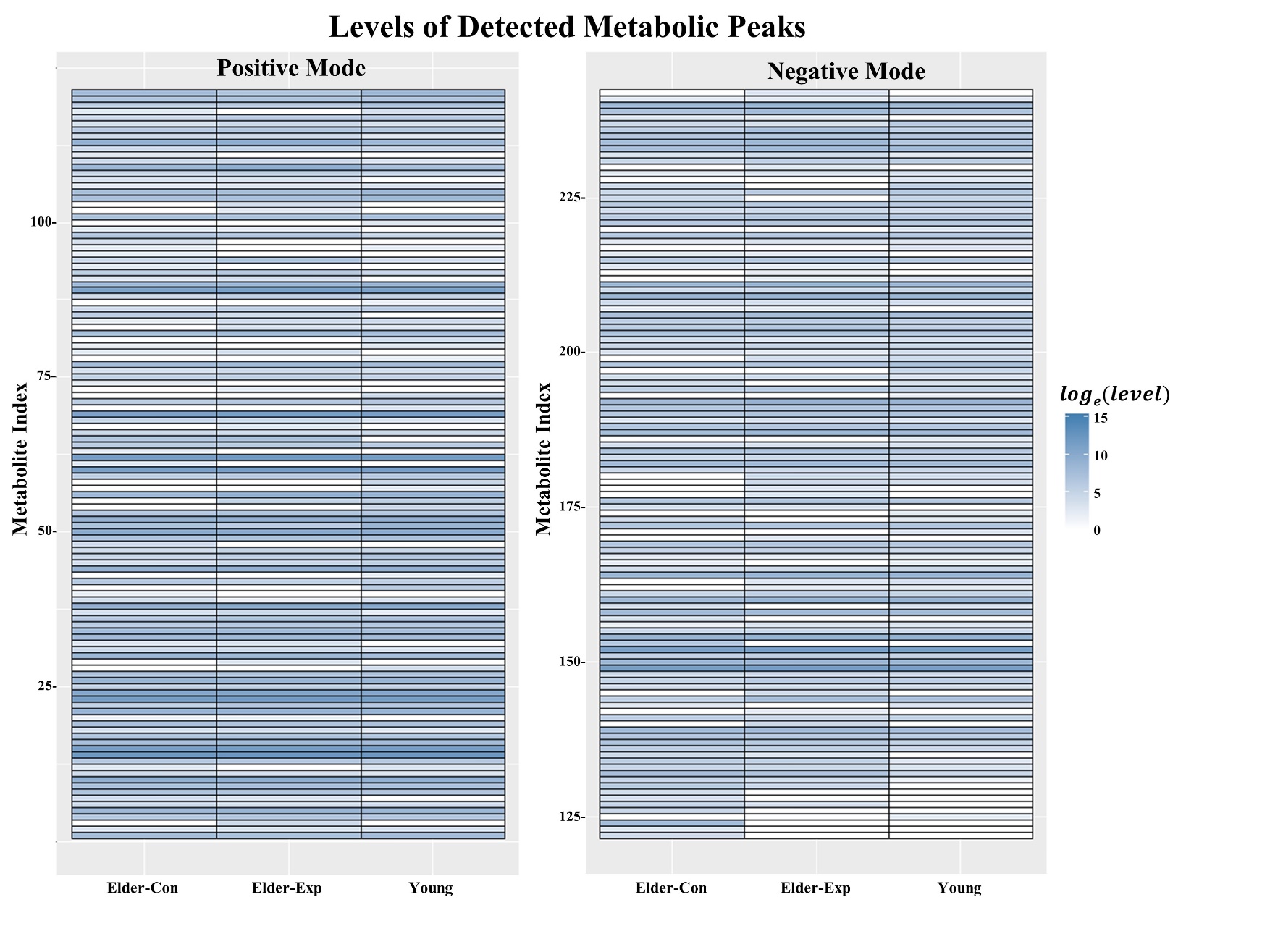


**Figure S-1.** Levels of detected metabolic peaks.
